# Hepatic progenitor cells promote the repair of schistosomiasis liver injury by inhibiting IL-33 secretion in mice

**DOI:** 10.1186/s13287-021-02589-y

**Published:** 2021-10-21

**Authors:** Beibei Zhang, Xiaoying Wu, Jing Li, An Ning, Bo Zhang, Jiahua Liu, Langui Song, Chao Yan, Xi Sun, Kuiyang Zheng, Zhongdao Wu

**Affiliations:** 1 Jiangsu Key Laboratory of Immunity and Metabolism, Xuzhou Key Laboratory of Infection and Immunity, Department of Pathogenic Biology and Immunology, Xuzhou Medical University, Xuzhou, Jiangsu China; 2 Department of Parasitology of Zhongshan School of Medicine, Sun Yat-sen University, Guangzhou, Guangdong China; 3 Key Laboratory of Tropical Disease Control, Ministry of Education, Sun Yat-sen University, Guangzhou, Guangdong China; 4 Department of Gastroenterology, The Third Affiliated Hospital of Sun Yat-sen University, Guangzhou, Guangdong China; 5 Jiangxi Provincial Institute of Parasitic Diseases, Nanchang, Jiangxi China; 6 The Eighth Affiliated Hospital of Sun Yat-sen University, Shenzhen, Guangdong China

**Keywords:** Hepatic schistosomiasis, Hepatic progenitor cells, IL-33

## Abstract

**Background:**

Hepatic schistosomiasis, a chronic liver injury induced by long-term *Schistosoma japonicum* (*S. japonicum*) infection, is characterized by egg granulomas and fibrotic pathology. Hepatic progenitor cells (HPCs), which are nearly absent or quiescent in normal liver, play vital roles in chronic and severe liver injury. But their role in the progression of liver injury during infection remains unknown.

**Methods:**

In this study, the hepatic egg granulomas, fibrosis and proliferation of HPCs were analyzed in the mice model of *S. japonicum* infection at different infectious stages. For validating the role of HPCs in hepatic injury, tumor necrosis factor-like-weak inducer of apoptosis (TWEAK) and TWEAK blocking antibody were used to manipulate the proliferation of HPCs in wild-type and IL-33^−/−^ mice infected with *S. japonicum*.

**Results:**

We found that the proliferation of HPCs was accompanied by inflammatory granulomas and fibrosis formation. HPCs expansion promoted liver regeneration and inhibited inflammatory egg granulomas, as well as the deposition of fibrotic collagen. Interestingly, the expression of IL-33 was negatively associated with HPCs’ expansion. There were no obvious differences of liver injury caused by infection between wild-type and IL-33^−/−^ mice with HPCs’ expansion. However, liver injury was more attenuated in IL-33^−/−^ mice than wild-type mice when the proliferation of HPCs was inhibited by anti-TWEAK.

**Conclusions:**

Our data uncovered a protective role of HPCs in hepatic schistosomiasis in an IL-33-dependent manner, which might provide a promising progenitor cell therapy for hepatic schistosomiasis.

**Supplementary Information:**

The online version contains supplementary material available at 10.1186/s13287-021-02589-y.

## Introduction

Schistosomiasis is one of the main human helminth infections that are responsible for liver injury. China is the main epidemic area of schistosomiasis, and there were approximately 30,170 patients with advanced schistosomiasis in 2019 [1, 2]. The most common pathological consequences of schistosomiasis are associated with chronic disease characterized by a predominant Th2 immune response to the constant stimulation of soluble egg antigen [3]. Thus, a granulomatous response is initiated by inflammatory cells’ infiltration around trapped eggs, which could induce secondary fibrosis formation. Meanwhile, hepatocyte apoptosis and necrosis inevitably occurred at the same time. Without efficient treatment, hepatic schistosomiasis can result in morbidity and even mortality [1]. Although the mechanisms underlying repair of liver injury in schistosomiasis have been studied in recent years, it remains largely unknown.

Adult stem/progenitor cells are vital in maintaining organ homeostasis under chronic pathological conditions. The stem/progenitor cell therapy has been largely studied and is likely to be a promising treatment for multiple organ injuries [4]. Many studies reported that the evolution of HPCs not only contributed to the regenerative and reparative response, but also inevitably correlated with the fibrotic process. The functions of HPCs in many human hepatic diseases or mouse models have been investigated [5]. However, to date, it is unclear whether HPCs modulate the liver injuries induced by *S. japonicum* infection.

The activation of HPCs is associated with the degree of inflammation and fibrosis. It is well known that the expansion and differentiation of HPCs are dependent on interaction with hepatic stellate cells (HSCs), macrophages, and extracellular matrix (ECM) [6]. Notably, macrophages and HSCs have been reported to be two key cellular components that contribute to the inflammatory granulomas, collagen fibers deposition, and the formation of fibrosis in schistosomiasis [7, 8]. All these indicate that HPCs can probably be activated in the process of this infectious disease. This study aimed to clarify the role and mechanism of HPCs in liver pathological regulation and repair. We found that the expansion of HPCs suppressed the development of hepatic granulomas and fibrosis in infected mice. Moreover, IL-33 was involved in HPCs-mediated liver injury repair.

## Materials and methods

### Mice model

Six- to eight-week-old male BALB/c mice were purchased from the Experimental Animal Center of Guangdong Province. They were supplied with sterile food and water ad libitum and housed in the Biosafety Level-2 (BSL-2) laboratory of Sun Yat-sen University. The IL-33^−/−^ mice with BALB/c background were generated with CRISPR/Cas9 technology. Briefly, two gRNAs matching the sequences flanked the exon 3. The gRNA sequences and Cas9 mRNA were co-microinjected into zygotes. The detailed strategy and the knockout sequence are shown in Additional file 1A. All mice were housed at the Specific Pathogen Free (SPF) facility with 21–26 °C room temperature, 40–70% humidity, and 12-h light/dark cycle in the animal center of Xuzhou Medical University. The genotypes of mice were measured before being infected with *S. japonicum* (Additional file 1B and 1C). All the procedures of animal experiments and reporting follow the ARRIVE guidelines.

We purchased the *Oncomelania hupehensis* (*O.hupehensis*) snails infected with cercariae from the National Institute of Parasitic Diseases, Chinese Center for Disease Control and Prevention in Shanghai. *O. hupehensis* snails were put into water without chlorine under light to release cercariae. To infect mice with cercariae, 15 ± 2 cercariae were picked onto a glass slide under the anatomical microscope and the glass was placed on the abdomen of a mouse for 15 min. After 4, 6, 10, and 14 weeks, control mice without infection and infected mice were killed under deep anesthesia with 2% pentobarbital at a dose of 45 mg/kg.

After 4 weeks of infection, for activating HPCs in vivo, mice were injected with 20 μg/kg body weight  of the recombinant tumor necrosis factor-like weak inducer of apoptosis (TWEAK) (R&D,1237-TW/CF, Minneapolis, Minnesota, USA) or phosphate-buffered saline (PBS) as control via tail intravenous injection. For the inhibition of the activation of HPCs, 2 mg/kg body weight TWEAK blocking antibody (Biolegend, 120,008, San Diego, CA, USD) or 2 mg/kg body weight isotype control antibody (Biolegend, 400,427, San Diego, CA, USD) was injected via tail intravenous injection. All doses used were followed the manufacturer’s instructions, and all injections were conducted twice a week for two weeks or five weeks.

### Serum albumin and IL-33 detection

Blood was collected and rested at room temperature for 2 h. After centrifuging at 4000 rpm for 10 min, the serum was separated and transferred to other tubes. One part with 200 μl was sent to KingMed Diagnostics Co., Ltd. (Guangzhou, China) for measuring the concentration of albumin. For the detection of IL-33 in serum, an enzyme-linked immunosorbent assay (ELISA) kit was used and obtained according to the manufacturer’s instructions (Invitrogen, 88–7333-22, Massachusetts, USA).

### Liver pathology measurement, immunohistochemistry and immunofluorescent analysis

The liver tissues were cut into 2 × 2 × 0.3 cm^3^ and fixed in 4% paraformaldehyde according to the standard protocols. Then, samples were cut into 4-μm-thick continuous sections. Hematoxylin and eosin (H&E) staining and Masson's trichrome staining were used to evaluate the percents of granulomas and fibrosis area, respectively. All these sections were scanned by a Zeiss Axio Scan.Z1 microscope (Carl Zeiss AG, Oberkochen, Germany), and the whole image of each section obtained was analyzed by Image Pro Plus 6.0 software.

HPCs proliferation was assessed by immunohistochemistry (IHC). In detail, all de-waxed sections were kept in boiling citrate solution for half an hour. After blocking the endogenous peroxidase activity with methanolic hydrogen peroxide (3%) for 10 min at room temperature, sections were incubated with 1% BSA for 30 min for blocking nonspecific antigen epitopes and incubated with primary A6 antibody (obtained from Developmental Studies Hybridoma Bank), CK19 antibody (Abcam, ab52625, Cambridge, UK), Epcam antibody (Abcam, ab213500, Cambridge, UK), F4/80 (CST, #30325, Darmstadt, Germany) antibody and CD45 (Proteintech, 60287-1-Ig, Wuhan, China) antibody at 4 °C overnight. After washing three times with PBS, 50 μl of ready-to-use HRP-labelled anti-mouse/rabbit general secondary antibodies (Dako, Copenhagen, Denmark) was added to each section and incubated for half an hour at room temperature. Finally, each section was observed under a light microscope when 3,3′-diaminobenzidine (DAB) substrate was added, and the reaction was stopped by putting the section into distilled water for 6 min. After sections were counterstained with hematoxylin and dehydrated, sections were mounted with neutral gum. For assessing the HPCs compartment, all sections were scanned by a Zeiss Axio Scan.Z1 microscope (Carl Zeiss AG, Oberkochen, Germany) to get the image of the whole tissue (original magnification, × 100). The positive HPCs within the bile/reactive ductules and the solitary or clumped A6- or CK19- or Epcam-positive HPCs that were present at the portal interface or in the parenchyma were counted. But cholangiocytes locating on the side of the lumen of the interlobular bile ducts were excluded from the counts. The number of HPCs was shown as the number of CK19- or Epcam-positive cells per field. For each group, at least four samples were analyzed [9, 10].

For immunofluorescent co-staining, mice livers were fixed in 4% paraformaldehyde overnight, and then, they were frozen in 22-oxacalcitriol (OCT) compound. The frozen sections were incubated with A6 antibody, SOX9 antibody (Abcam, Ab185230, Cambridge, England), and HNF4α antibody (Abcam, Ab41898, Cambridge, England) overnight at 4 °C. After washing with PBS three times, slides were incubated with appropriate secondary antibodies for 1 h at room temperature. Fluorescent mounting media containing DAPI were added to the sections for nuclear staining and mounting. Pictures were captured by a confocal microscope (Leica, Delaware, USA). And numbers of double-positive cells were analyzed.

### TUNEL assay

The apoptosis of hepatocytes was detected by TUNEL staining with an In Situ Cell Death Detection Kit (Roche, Switzerland). All the procedures were performed strictly according to the manufacturer’s instructions. Briefly, paraformaldehyde-fixed tissue sections were dewaxed and subsequently incubated with proteinase K at 27 °C for 25 min. After washing three times, permeabilization solution containing 0.1% Triton X–100 and 0.1% sodium citrate was added and kept for 8 min. Then, a 50 μl TUNEL reaction mixture was added. 50 μl label solution and DNase I recombinant were added in the negative control and positive control, respectively. All samples were incubated for 60 min at 37 °C in a humidified incubator in the dark. Lastly, one drop of DAPI was added and kept for 5 min. After washing three times with PBS, all slides were covered with an anti-fluorescence quenching agent and captured under an Olympus BX-63 microscopy (Olympus, Tokyo, Japan). For each group, at least four samples were analyzed.

### Hydroxyproline

Liver tissues (40–50 mg) were weighed precisely for measuring the content of hydroxyproline. All the procedures followed the instructions of a commercial hydroxyproline assay kit (Jiancheng Institute of Biotechnology, A030-2, Nanjing, China). The OD value of each sample was read by a Tecan Sunrise system (Sunrise, TECAN, Männedorf, Austria), and the content of hydroxyproline was calculated according to the formula listed in the instructions.

### RNA extraction, cDNA synthesis, and real-time PCR

Total RNA was isolated from the liver tissue using Trizol reagent (Invitrogen, New York, USA) following the manufacturer’s protocols. The quantity and quality of the RNA were estimated by a NanoDrop 2000 spectrophotometer (Thermo Fisher Scientific, 78441, Massachusetts, United States). Then, 3 μg RNA was reverse-transcribed using a cDNA Synthesis Kit (Thermo Fisher Scientific, 78441, Massachusetts, USA). The expression levels of *HGF* mRNA, *IL-6* mRNA, *α-SMA* mRNA, *Collagen-I* mRNA, *IL-33* mRNA, *Albumin* mRNA, and *GAPDH* mRNA were detected using SYBR Premix Ex TaqTM (TaKaRa, Osaka, Japan), and the specific primer pairs are listed in Table 1. The reaction procedure was followed as described previously [11] and performed with a CFX 96 touch instrument (Bio-Rad, CA, USA). The *GAPDH* mRNA level was used for normalization. All the relative mRNA levels were calculated using the 2^−ΔΔCT^ method and normalized to the control group.

**Table 1:** Primers used for real-time PCR

Genes	Primer	Sequence(5′–3′)
*GAPDH*	Forward primer	ACTCCACTCACGGCAAATTC
	Reverse primer	TCTCCATGGTGGTGAAGACA
*HGF*	Forward primer	CCAGAGGTACGCTACGAAGT
	Reverse primer	CTGTGTGATCCATGGGACCT
*IL-6*	Forward primer	GACTGATGCTGGTGACAACC
	Reverse primer	AGACAGGTCTGTTGGGAGTG
*α-SMA*	Forward primer	CACAGCCCTGGTGTGCGACAAT
	Reverse primer	TTGCTCTGGGCTTCATCCCCCA
*Collagen-I*	Forward primer	TCCTGCGCCTAATGTCCACCGA
	Reverse primer	AAGCGACTGTTGCCTTCGCCTC
*IL-33*	Forward primer	ACACAGTCTCCTGCCTCCCT
	Reverse primer	CCACACCGTCGCCTGATTGA
*Albumin*	Forward primer	GCCCACTGTCTTAGTGAGGT
	Reverse primer	ATACAAGAACGTGCCCAGGA

### Western blotting

Total protein was extracted from 30 to 50 mg liver tissue using an extraction buffer containing RIPA lysis buffer (Thermo Fisher Scientific, 89901, Massachusetts, USA), and protease and phosphatase inhibitors cocktails (Thermo Fisher Scientific, 78441, Massachusetts, USA). A total of 50 μg protein was separated on an SDS–polyacrylamide gel with 120 V for 2 h and transferred to a PVDF membrane with 100 mA for 3 h on ice. Then, the PVDF membrane was blocked in 5% nonfat milk in TBST for 2 h at room temperature and incubated with mouse anti-IL-33 antibody using at 1:1000 dilution (Abcam, ab54385, Cambridge, England) and mouse anti-GAPDH antibody using at 1:2000 dilution (Sigma-Aldrich, G8795, St. Louis, MO, USA) overnight at 4 °C. Anti-mouse IgG (CST, 7076S, Darmstadt, Germany) was diluted at a 1:5000 ratio and used as a secondary antibody. In Fig. 7A, the secondary antibody was diluted at a 1:20,000 ratio for reacting with the primary mouse anti-IL-33 antibody. After washing 3 times with TBST, the membrane was incubated with a secondary antibody for 1 h at room temperature. After adding ECL substrate (Millipore, MA, USA), the protein band was visualized and captured with an Amersham Imager 600 system (GE, CT, USA). The exposure time was optimized as much as possible. The density of the band was quantified by Image J2x software and normalized to GAPDH.

### Isolation of hepatocytes, HSCs, and macrophages

Livers were perfused with 37 °C PBS at a rate of 2 ml/min for 5 min to flush the blood out of the livers and digested in situ subsequently with 37 °C 0.04% collagenase type IV (Gibco, Massachusetts, USA) at a rate of 2 ml/min for 8 min. Then, livers were removed and tore into pieces. After keeping digesting for 6 min at 37 °C, the digestion was added cold DMEM containing 20% FBS to stop digesting and filtered through a 70-μm membrane into a 50-ml tube. The supernatant was centrifuged at 500 g for 5 min at 4 °C. The cell pellet was resuspended and centrifuged at 50 g for 4 min. The hepatocytes sunk to the bottom of the tube, and nonparenchymal cells remained in the supernatant. The supernatant was recentrifuged at 500 g for 7 min, and the supernatant was removed carefully. The cell pellet was added 12% and 18% iodixanol gradient (OptiPrep; Axis-Shield PoC AS, Oslo, Norway) and centrifuged at 1400 g without brakes for 20 min. So, we can get the HSCs and macrophages. To get high purified cells, fluorescence-activated cell sorting was used. The CD11b-positive cells were referred to as macrophages and negatives cells as HSCs. Cells were obtained by FACS-Calibur sorter (BD, New Jersey, USA).

### Statistical analysis

Quantitative data were shown as Mean ± SEM. Statistical significance was analyzed with SPSS 19.0 software. Comparisons between two groups were determined with independent-sample *t*-test, and significances between multiple groups were determined by one-way ANOVA followed by LSD or nonparametric test followed by the Kruskal–Wallis H test. *P* < 0.05 was considered as statistically significant.

## Results

### Liver regeneration is involved in the alleviation of liver injury induced by *S. japonicum* infection

**Fig. 1:**
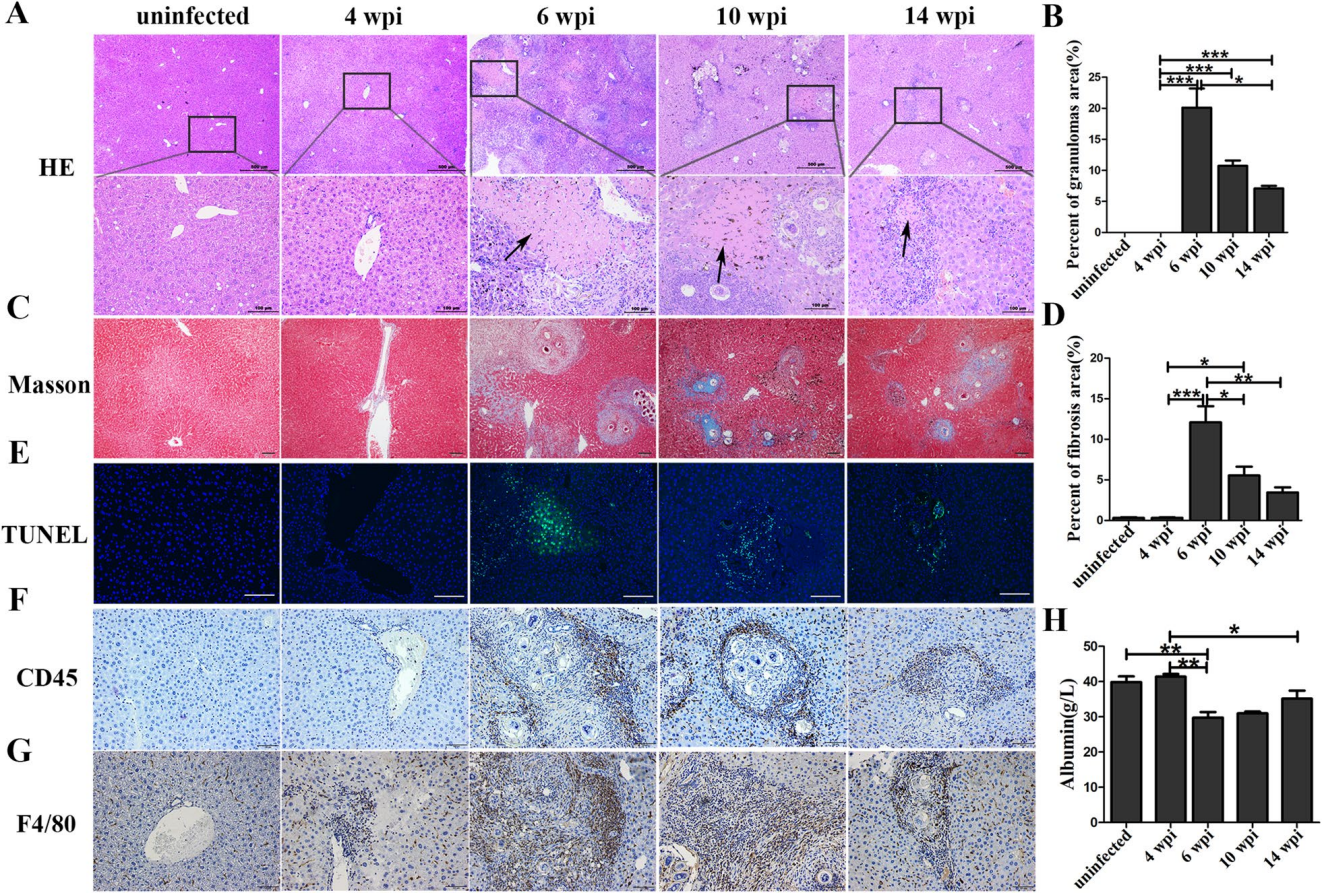
The dynamic of histopathological changes and liver regeneration in a single infection mouse model with *S. japonicum*. **A** Representative images of HE staining at different infection time points. Arrows indicate the necrosis. **B** The statistical analysis of percent of granulomas area. *n* = 7 in uninfected group, *n* = 5 in each infected group. **C** Representative images of Masson staining. **D** The statistical analysis of percents of fibrosis area. *n* = 7 in uninfected group, *n* = 5 in each infected group. **E** Representative images of TUNEL staining. *n* = 3 mice/group. **F** Representative images of CD45 staining. *n* = 3 mice/group. **G** Representative images of F4/80 staining. *n* = 3 mice/group. **H** The expression level of albumin in serum. *n* = 7 in uninfected group, *n* = 5 mice in each infected group. Images were captured by an inverted microscope (Olympus, BX63, Tokyo, Japan). *P < 0.05, **P < 0.01, ***P < 0.001

To investigate whether liver regeneration occurred in mice following infection, liver samples were collected at different stages of infection. By HE and Masson‘s staining, we found that the liver showed slightly inflammatory cells infiltration and fibrotic formation at 4 weeks post-infection (wpi). However, inflammatory granulomas and fibrosis area increased sharply at 6 wpi (*P* < 0.001). Interestingly, the inflammatory granulomas and fibrosis area started to decrease from 10 to 14 wpi (Fig. 1A–D). Meanwhile, there were no necrosis and apoptotic hepatocytes at 4 wpi. Large numbers of necrotic cells and apoptotic hepatocytes appeared around the granulomas at 6 wpi but reduced significantly after 10 wpi (Fig. 1E). This pattern was similar to the inflammatory and the fibrotic status. The CD45-positive inflammatory cells (Fig. 1F) and F4/80-positive (Fig. 1G) cells also increased significantly at 6 wpi and decreased after 10 weeks of infection. All these implied that the liver histopathology, including inflammatory granulomas and fibrosis, could be self-recovery. Notably, compared with the uninfected and 4 wpi groups, the level of hepatocyte marker albumin in blood decreased significantly at 6 wpi. However, with the liver pathology relieving after 10 wpi, the level of albumin increased gradually (Fig. 1H). Thus, we suspected that liver regeneration might be involved in this process.

### The expansion of HPCs was induced in mice with *S. japonicum* infection

**Fig. 2:**
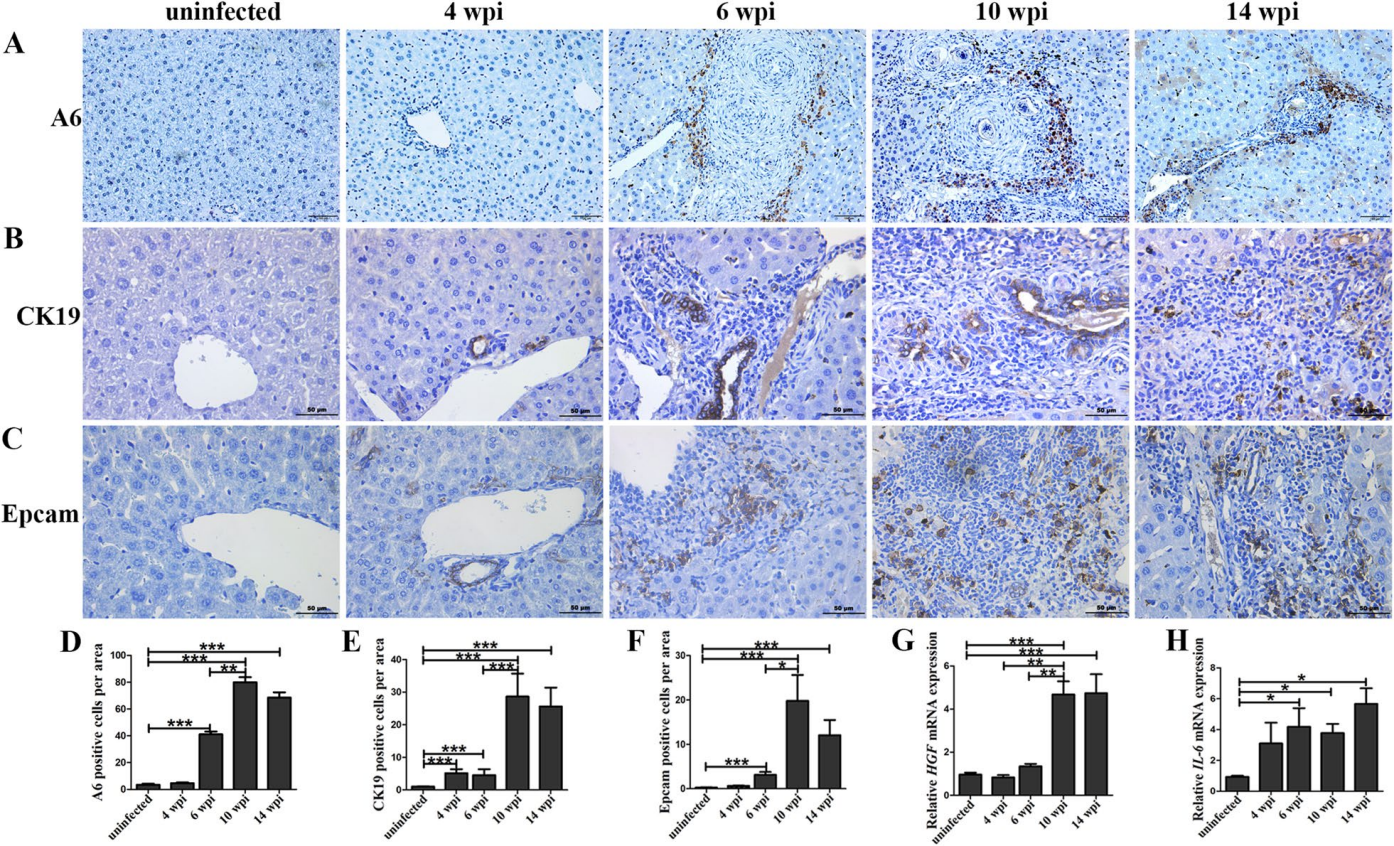
The expansion of HPCs was induced in mice with *S. japonicum* infection*.* Representative images of A6 staining (**A**), CK19 staining (**B**), and Epcam staining (**C**). The statistical analysis of A6-positive cells per area (**D**), CK19-positive cells per area (**E**), and Epcam-positive cells per area (**F**). *n* = 4 mice/group. **G** Relative *HGF* mRNA level in livers. *n* = 4 mice/group. **H** Relative *IL-6* mRNA level in livers. *n* = 4 mice/group. *P < 0.05, **P < 0.01, ***P < 0.001

Following liver injury, HPCs have been reported to play potential roles in liver repopulation and regeneration [12]. Thus, we asked whether HPCs contributed to self-recovery via promoting liver regeneration in hepatic schistosomiasis or not. Markers of HPCs A6, CK19, and Epcam were detected by IHC. As shown in Fig. 2A, and Fig. 2D, the level of A6 was higher in the infected group than that observed in the uninfected group, and it remained high level until 14 wpi (*P* < 0.001). In addition, CK19 and Epcam also increased at 6 wpi (*P* < 0.001) and kept increasing after 10 wpi (Fig. 2B, C, E, F). We also checked the HPCs-related cytokines *HGF* mRNA (Fig. 2G) and *IL-6* mRNA (Fig. 2H) expression profiles. The mRNA of *HGF* upregulated sharply at 10 wpi and kept at a high level at 14 wpi, and *IL-6* mRNA was higher than the uninfected group at 6 wpi and lasted for 14 wpi. These data indicated that, with the inflammatory granulomas and fibrosis formation, the activation and proliferation of HPCs occurred.

### HPCs promoted the repair of liver injury induced by *S. japonicum* infection

**Fig. 3:**
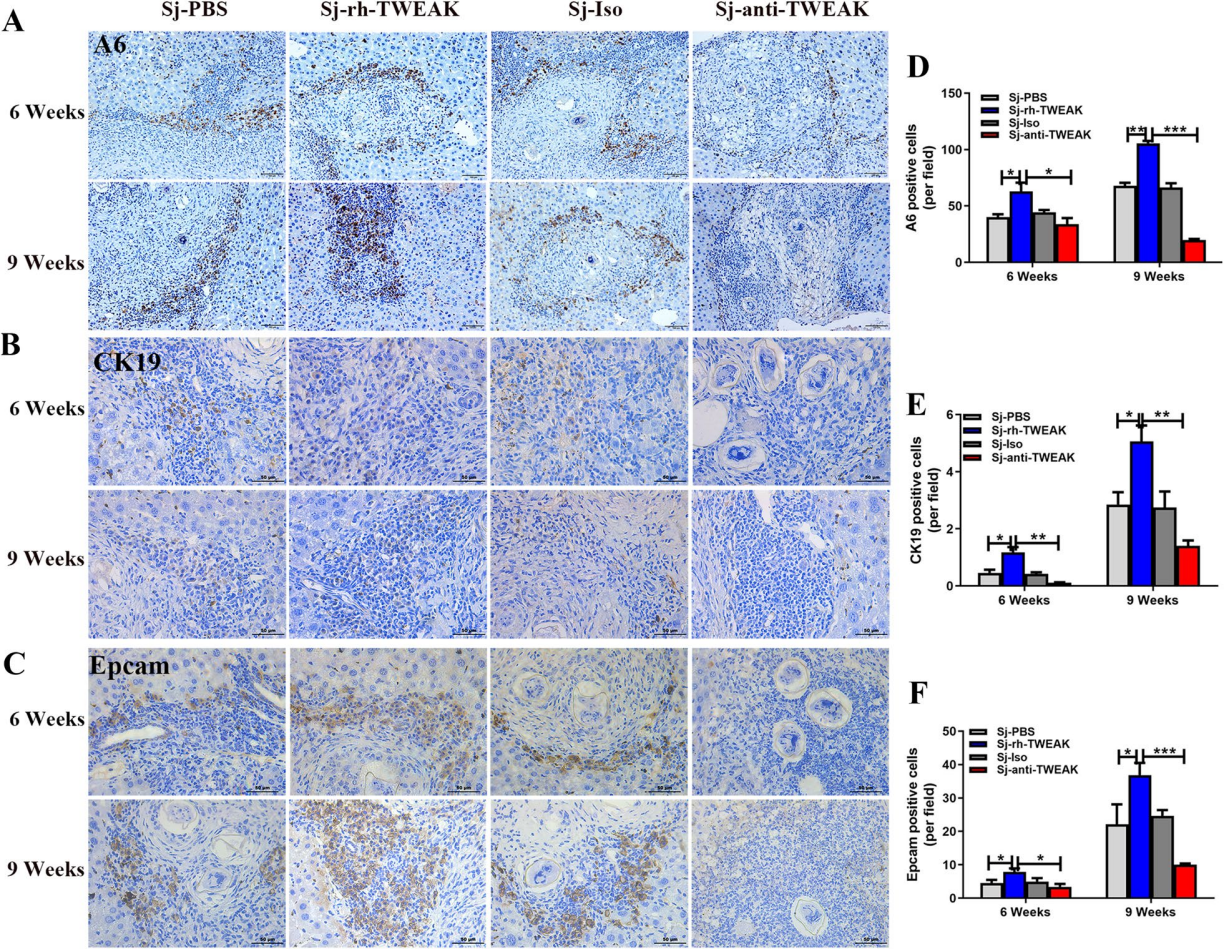
TWEAK induced the expansion of HPCs in infected mice model*.* Representative images of A6 staining (**A**), CK19 staining (**B**), and Epcam staining (**C**). The statistical analysis of A6-positive cells (**D**), CK19-positive cells (**E**), and Epcam-positive cells (**F**) per area in the four groups. *n* = 3 mice/group. *P < 0.05, **P < 0.01, ***P < 0.001

To clarify whether HPCs mediate liver regeneration and thus modulate the repair of liver injury in *S. japonicum* infected mice or not, TWEAK, a specific mitogen for the proliferation of HPCs, was used to experimentally manipulate the proliferation of HPCs. TWEAK-neutralizing antibody was used to inhibit the proliferation of HPCs. PBS and isotype antibody were used as control. At 6 wpi, there were no apparent differences in the expression of A6, CK19, and Epcam between the isotype and anti-TWEAK treatment mice with infection (Fig. 3A–F). All these markers were higher in the rh-TWEAK-treated group than the PBS and anti-TWEAK-treated groups. At 9 wpi, by comparing with the infected group with PBS treatment, A6 (*P* < 0.01), CK19 (*P* < 0.05), and Epcam (*P* < 0.05) were higher in the rh-TWEAK treated group. Conversely, A6 (*P* < 0.001), CK19 (*P* < 0.01), and Epcam (*P* < 0.001) decreased significantly in the anti-TWEAK group. These data approved that rh-TWEAK induced the activation and proliferation of HPCs while blocking antibody-inhibited HPCs response successfully.

**Fig. 4:**
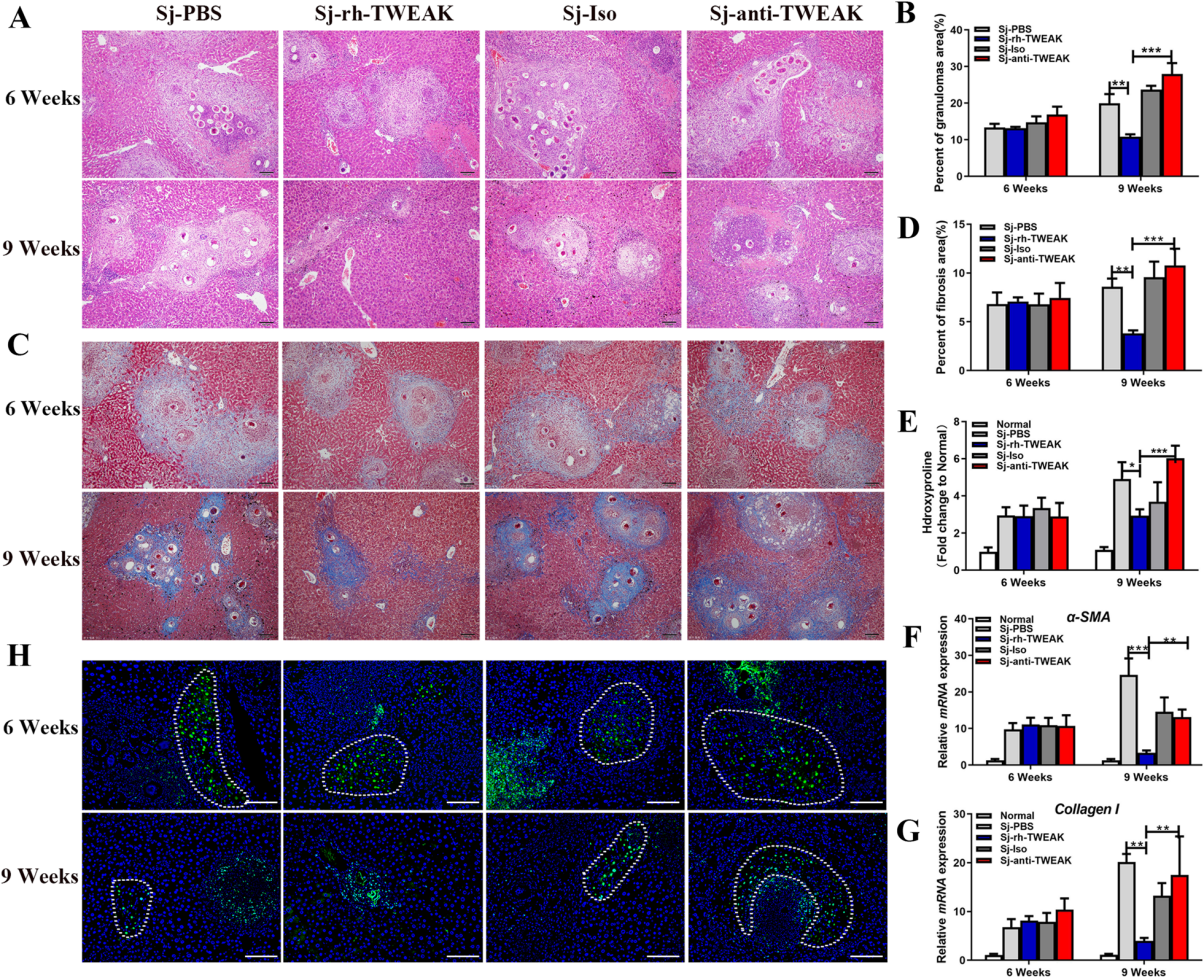
The expansion of HPCs promoted the repair of liver injury induced by *S. japonicum* infection*.* **A** Representative images of HE staining. **B** The statistical analysis of percents of granulomas area. *n* = 5 mice/group **C** Representative images of Masson's staining. **D** The statistical analysis of percents of fibrosis area. *n* = 5 mice/group. **E** The content of hydroxyproline in mice liver. *n* = 5 mice/group. Real-time PCR analysis of *α-SMA* mRNA (**F**), and *Collagen-I* mRNA (**G**) in mice liver. n = 5 mice/group. **H** Representative images of TUNEL staining. *n* = 5 mice/group. *P < 0.05, **P < 0.01, ***P < 0.001

To address whether the proliferation of HPCs modulated the liver injury or not, HE and Masson’s staining were performed to determine the inflammatory granulomas and fibrosis (Fig. 4A–D), respectively. At 6 wpi, these parameters showed no apparent differences among these four infected groups. But at 9 wpi, in contrast to Sj-PBS and Sj-Iso groups, the percentage of granulomas area and fibrosis area was significantly decreased in infected livers with HPCs expansion. Conversely, it increased obviously in infected livers with a reduced expansion of HPCs. We also detected the content of hydroxyproline (Fig. 4E), pro-fibrotic genes α-SMA (Fig. 4F), and Collagen-I (Fig. 4G). They showed similar trends with HE and Masson's staining. Additionally, we found that the necrosis of hepatocytes was serious in these four infected groups at 6 wpi and alleviated at 9 wpi. It was relatively milder in the Sj-rh-TWEAK group and more severe in the Sj-anti-TWEAK group (Fig. 4A, H). All these data taken together indicate that the proliferation of HPCs can inhibit the formation of granulomas and fibrosis in *S. japonicum*-infected mice.

### HPCs promoted liver regeneration in *S. japonicum*-infected mice

**Fig. 5:**
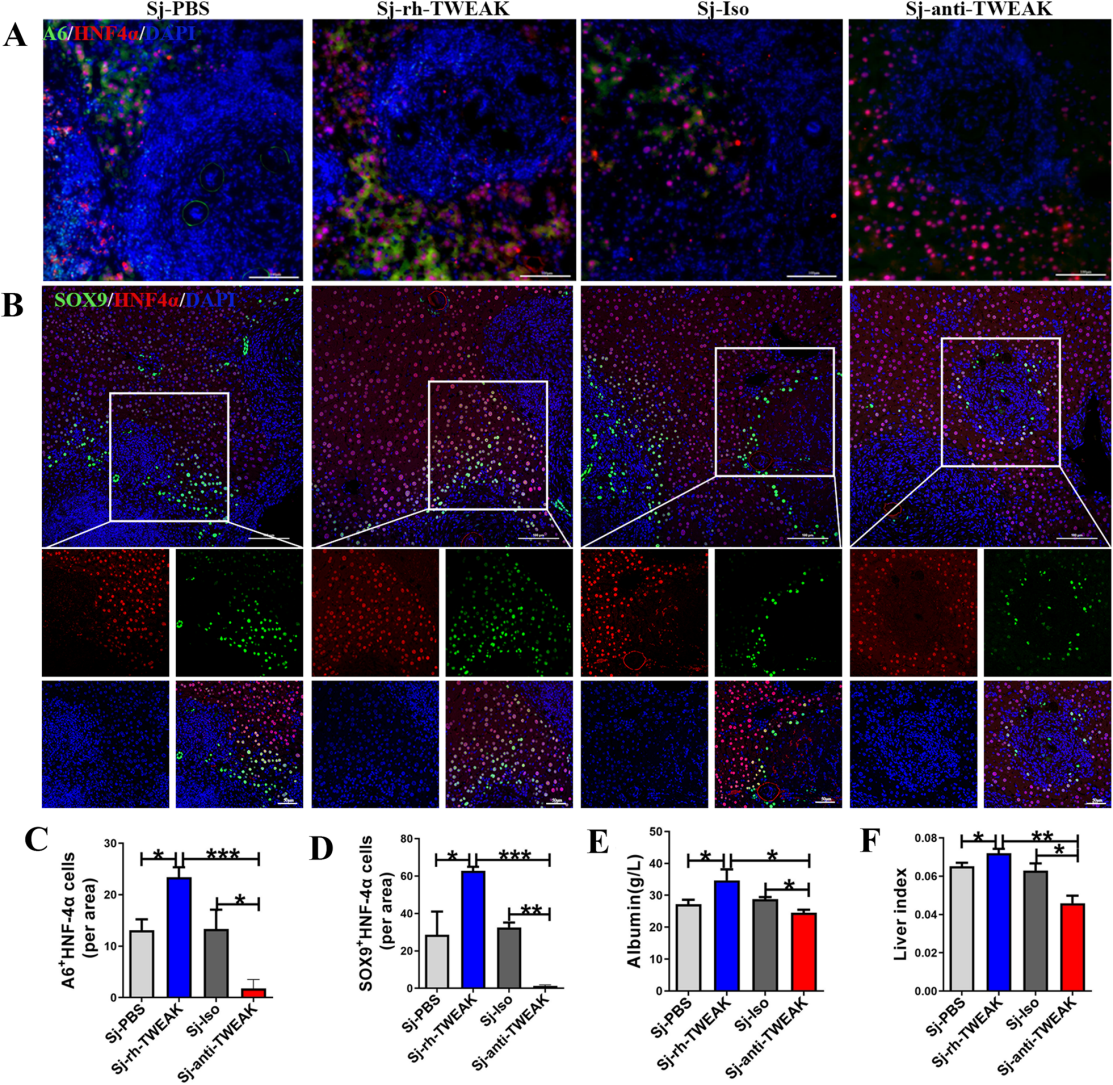
HPCs promoted liver regeneration in *S. japonicum* infection mice. **A** Representative double-immunostaining with A6/Hnf4α in mice liver. **B** Representative double-immunostaining with SOX9/HNF4α in mice liver. **C** The number of A6^+^HNF4α^+^ cells in (**A**) were qualified. *n* = 4 mice/group. **D** The number of SOX9^+^HNF4α^+^ cells in (**B**) were qualified. *n* = 4 mice/group. **E** The expression level of albumin in serum. *n* = 6 in Sj-PBS and Sj-rh-TWEAK group, *n* = 5 in Sj- Iso group, *n* = 4 in Sj-anti-TWEAK group. **F** The ratio of liver weight to body weight. *n* = 6 in Sj-PBS and Sj-rh-TWEAK group, *n* = 5 in Sj-Iso group, *n* = 4 in Sj-anti-TWEAK group. *P < 0.05, **P < 0.01, ***P < 0.001

As the proliferated HPCs could inhibit the hepatic schistosomiasis, we asked whether HPCs protected the liver from injury by facilitating liver regeneration. As results shown in Fig. 5A–D, some of the cells expressing HPCs markers A6 and SOX9 could also express hepatocytes marker HNF4α in Sj-PBS and Sj-Iso groups. These bi-phenotypic cells were more in mice with rh-TWEAK treatment (*P* < 0.05). But it could be hardly observed in mice with anti-TWEAK treatment. Additionally, it was found that the liver index (liver-to-body weight ratio) and serum albumin improved significantly in the rh-TWEAK-treated mice and decreased with anti-TWEAK treatment (Fig. 5E, F). However, the number of Ki67-positive hepatocytes was small and remained unchanged among all groups (Additional file 3). So, the replication of mature hepatocytes did not get involved in the repair of hepatic schistosomiasis. All these data indicated that proliferated HPCs could promote the liver regeneration in *S. japonicum*-infected mice.

### HPCs inhibited IL-33 secretion in *S. japonicum*-infected mice

**Fig. 6:**
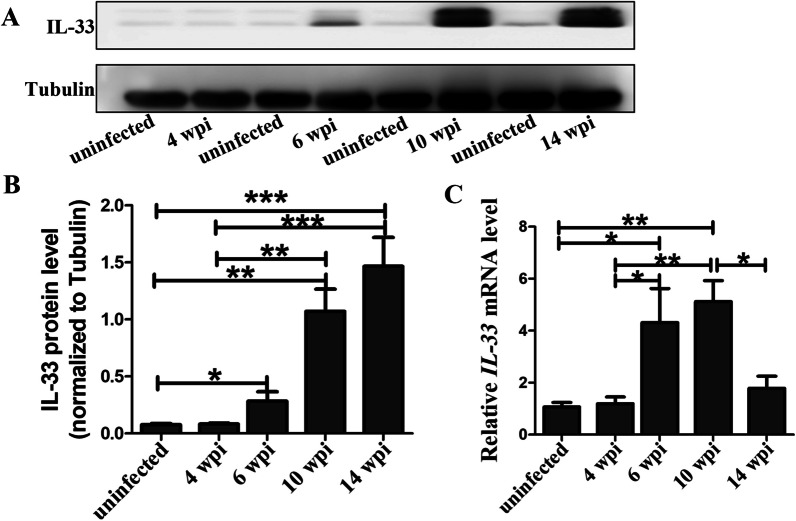
The dynamic change of IL-33 in *S. japonicum* infected mice model*.* **A** Representative bands of IL-33 protein by western blot. **B** The statistical analysis of the IL-33 protein. *n* = 4 mice/group. **C** Real-time PCR analysis of *IL-33* mRNA. *n* = 5 mice/group. *P < 0.05, **P < 0.01, ***P < 0.001

**Fig. 7:**
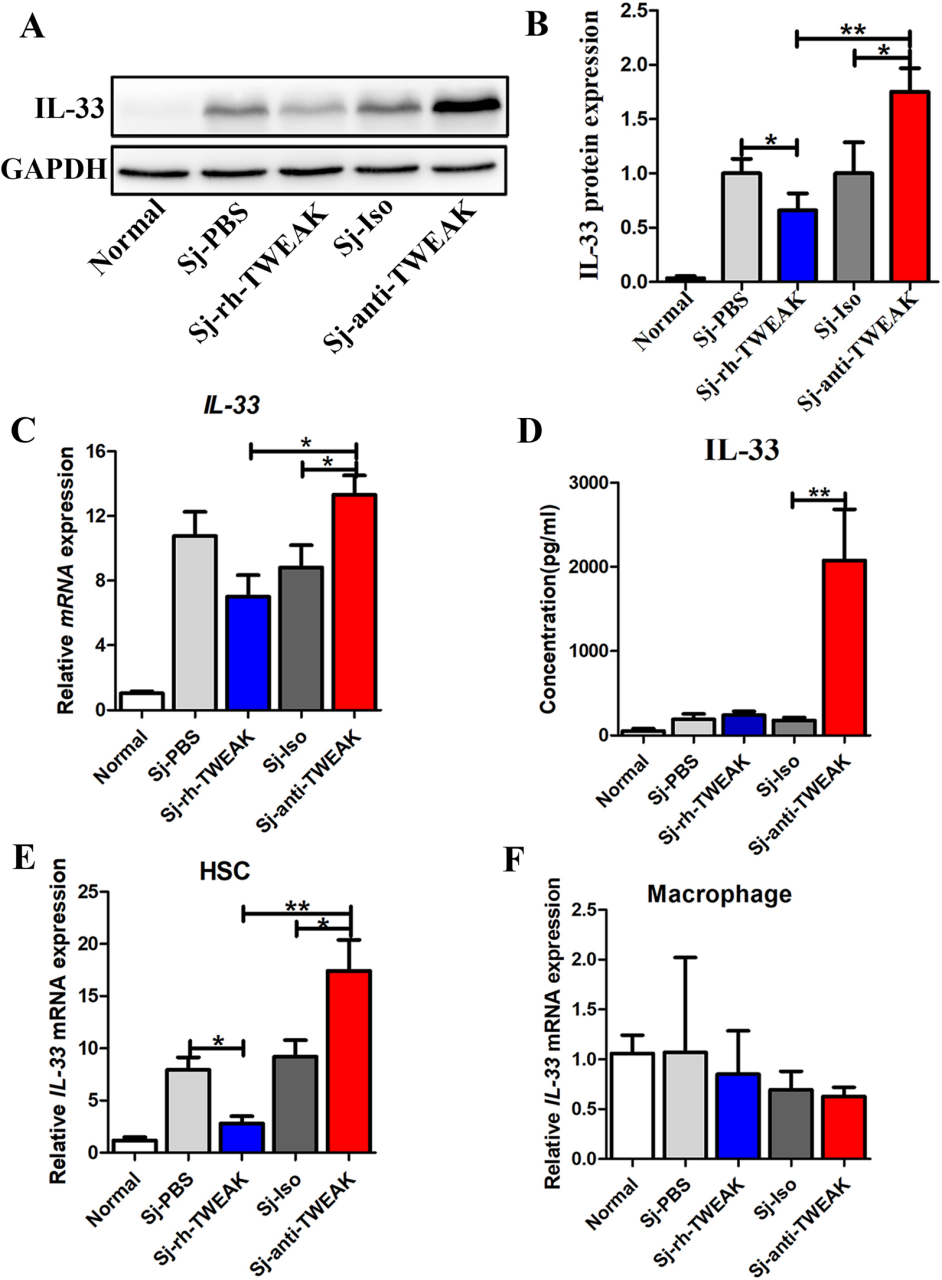
HPCs inhibited IL-33 secretion in the liver of *S. japonicum*-infected mice. **A** Representative bands of IL-33 protein by western blot in different groups. **B** The statistical analysis of the IL-33 protein. *n* = 4 mice/group. **C** Real-time PCR analysis of *IL-33* mRNA in the liver. *n* = 5 mice/group. **D** ELISA analysis of the concentration of *IL-33* in serum. *n* = 4 mice/group. **E** Real-time PCR analysis of *IL-33* mRNA in HSC. *n* = 4 mice/group. **F** Real-time PCR analysis of *IL-33* mRNA in macrophage. *n* = 4 mice/group.*P < 0.05, **P < 0.01

IL-33 is an important inflammatory cytokine, which can promote the hepatic fibrosis by mediating a shift to the Th2 paradigm [13]. In this single infection mouse model, it was found that the protein and mRNA levels of IL-33 elevated sharply at 6 wpi (Fig. 6). This high expression remained until 14 wpi. Then, we sought to clarify whether HPCs can modulate the secretion of IL-33 or not. Interestingly, promoting HPCs proliferation resulted in decreased expression of IL-33. In contrast, it was higher when the proliferation was inhibited (Fig. 7A–C). Besides, we detected the concentration of IL-33 in serum, which slightly increased in HPCs proliferated group, but strikingly augmented when HPCs proliferation was inhibited (Fig. 7D, P < 0.01). This finding suggested that the HPCs’ role in promoting the repair of hepatic schistosomiasis might be through inhibiting IL-33 secretion.

IL-33 was identified to be produced by some endothelial cells, epithelial cells, and hematopoietic cells including macrophages [14]. HSCs and macrophages are two major cellular components contributing to liver injury in schistosomiasis japonica [3]. In our present study, HSCs and macrophages were isolated from normal and infected livers. The ratio of HSCs is 77.75% by 12% iodixanol isolation (Additional file 2A), and macrophages are 64.12% by 18% iodixanol isolation (Additional file 2B). As shown in Additional files 2C and 2D, the isolated hepatocytes expressed a high level of albumin. But the expression of albumin is too low to be detected in HSCs and macrophages. The *α-SMA* mRNA is only high in HSCs but not in macrophages and hepatocytes. All these suggested that these three cell types were isolated successfully with high purities. Then, the mRNA level of* IL-33* was determined. As the result shown in Fig. 7E, IL-33 could be detected in HSC isolated from liver of infected mice. But it decreased when HPCs were manipulated to proliferate (*P* < 0.05). In contrast, inhibiting the proliferation of HPCs upregulated the mRNA level of IL-33 in HSCs. However, the expression of IL-33 in macrophages was kept at a base level and showed no changes regardless of the population of HPCs (Fig. 7F). All these data indicate that the proliferation of HPCs inhibits the expression of IL-33, which especially in HSCs.

### HPCs alleviated the hepatic schistosomiasis by inhibiting IL-33 secretion

**Fig. 8:**
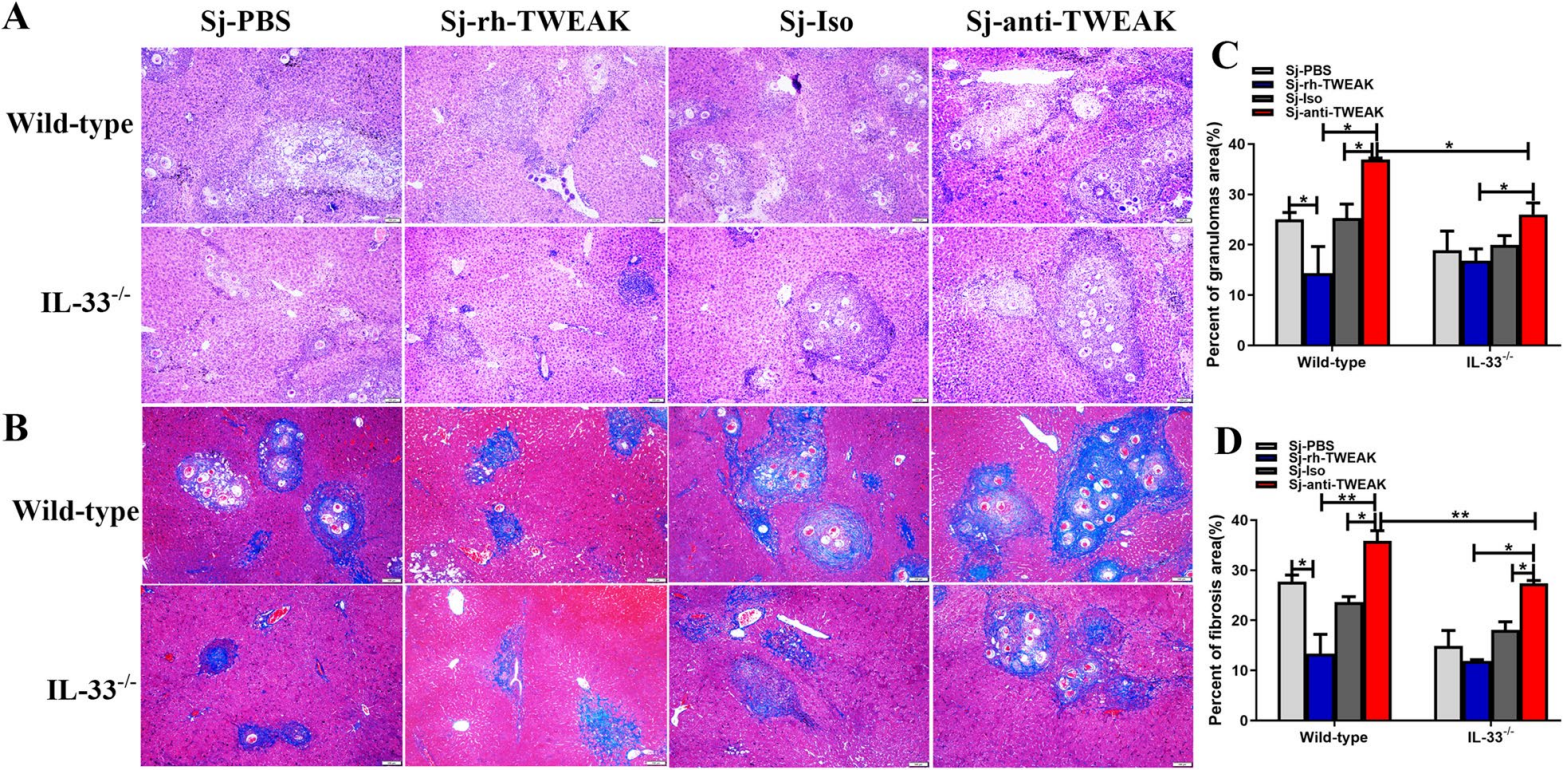
HPCs alleviated the hepatic schistosomiasis by inhibiting IL-33 secretion. **A** Representative images of HE staining in different groups. **B** The statistical analysis of percents of granulomas area. *n* = 3 mice/group. **C** Representative images of Masson's staining. **D** The statistical analysis of percents of fibrosis area. *n* = 3 mice/group. *P < 0.05, **P < 0.01

To clarify HPCs alleviate liver injury via modulating IL-33 expression, wild type and IL-33^−/−^ mice were infected with *S. japonicum*. As a result, there were no obvious differences in inflammatory granulomas and fibrosis between wild-type and IL-33^−/−^ mice liver with rh-TWEAK treatment. However, inflammatory granulomas and fibrosis were slighter in IL-33^−/−^ mice than wild-type mice when the proliferation of HPCs was inhibited by anti-TWEAK (Fig. 8). These indicated that HPCs weakened the liver injury by repressing IL-33 secretion in the livers of wild-type mice. So these pathologies showed no obvious differences in IL-33^−/−^ mice with HPCs proliferation. But, IL-33 was higher in wild-type mice when the HPCs proliferation was inhibited (Fig. 8, *P *< 0.05). Knocking out of IL-33 could also decrease liver injury significantly in mice with few HPCs. Thus, HPCs might alleviate the inflammatory granulomas and fibrosis via inhibiting IL-33 secretion.

## Discussion

The liver is special for its remarkable regenerative ability after tissue loss [15]. In a single mouse model of *S. japonicum* infection, we showed that the expansion of HPCs contributed to liver regeneration. Indeed, the expansion of HPCs inhibited the formation of inflammatory egg granulomas and secondary fibrosis. Moreover, we identified that the regulatory function of HPCs was through the inhibition of the secretion of IL-33. Thereby, we firstly revealed the role and mechanism of HPCs playing in hepatic schistosomiasis, which was important for the understanding of the pathogenic mechanism and developing a new strategy for this liver disease.

Chronic hepatic schistosomiasis results from long-standing infections with massive collagen deposition in the periportal space and tremendous inflammatory cells infiltration [1]. Though it can lead to unreversible fibrosis and even cirrhosis, the hepatocellular function remains nearly at a normal level [16]. In this study, we found that inflammatory granulomas, fibrosis, and hepatocytes injury cannot be observed before 4 wpi. But all these pathological changes outbroke at 6 wpi and ameliorated significantly with the lastingness of infection. It implied that liver injury induced by *S. japonicum* infection could repair itself.

Liver regeneration after tissue injury involves the supplement from several cell types [17]. With the development of regenerative medicine, a population of cells residing within the distal part of the bile duct (also called the canals of Hering) was identified as endogenous stem cells in the liver [18]. It possess the bipotential capacity to contribute to regeneration in the diseased liver. Notably, the mechanism of regeneration varies in different liver diseases and diseases at different stages [19, 20]. In conditions with steady status, the homeostasis is maintained by mature hepatocytes. But the regeneration was dominated by activated HPCs in the liver with prolonged injury or massive hepatocytes and cholangiocytes loss [21]. In the mouse model of *S. japonicum* infection, we found that the proliferation of HPCs upregulated after 6 wpi. It was no accident that the collagen deposition, CD45, and F4/80 all increased concurrently. These represented the activation of the HSCs, accumulation of macrophages, and liver inflammatory response. It was well studied that HSCs and infiltrated macrophages were important determinants of HPCs’ expansion [22, 23]. They can provide pro-mitogenic factors such as HGF, TGF-β, TNF-α, and IL-6 [21]. More importantly, activation of the Notch signaling pathway by HSC and Wnt/β-catenin signaling pathway was vital for the differentiation of HPCs [24, 25]. In contrast to 10 wpi, HPCs increased slightly at 6 wpi, which was an acute stage for an infection in mice. Therefore, HPCs might take part in the repair of hepatic injury at the chronic stage.

To confirm the function of HPCs in hepatic schistosomiasis, TWEAK, a specific mitogen for HPCs, was used to manipulate the proliferation of HPCs. We found that the expansion of HPCs alleviated the inflammatory granuloma and fibrosis efficiently. Additionally, bi-phenotypic cells expressing HPCs markers A6 or SOX9 and hepatocyte marker HNF4α could be observed. Besides, liver index and albumin increased significantly with HPCs’ expansion. Interestingly, the proliferation of mature hepatocytes was not affected by HPCs. Thus, HPCs but not hepatocytes promoted liver regeneration in *S. japonicum*-infected mice liver. Even though rh-TWEAK was injected at 4 wpi, the expansion of HPCs could not inhibit the progress of liver injury at 6 wpi. We deemed that HPCs could not define against the drastic pathogenicity of eggs at the acute stage. But with the continuous expansion of HPCs induced by rh-TWEAK, they can effectively prevent liver injury at 9 wpi.

Professor Xu and his colleagues reported that mesenchymal stem cells could ameliorate fibrosis in infected mice [26]. Although MSCs have been reported to be used in the clinic for several cases, the samples size of MSCs on liver transplantation for clinical trials is insufficient. To date, there are several main obstacles for MSCs’ application in liver transplantation: ethical doubt, safety concerns, systemic immunosuppression, sources of MSCs, time delays, and high costs for the generation and handling of MSCs [27, 28]. Besides, the truth is that most of the patients with schistosomiasis live in remote and economically backward regions [16]. It is too expensive for them to receive liver transplantation with stem cells. Our data suggested that specific mitogen TWEAK could facilitate the proliferation of liver endogenous stem/progenitor cells HPCs. Proliferated HPCs could promote the repair of the liver injury sufficiently. Therefore, our work might supply a potentially effective and economical therapy for schistosomiasis.

IL-33, a member of the IL-1 family, plays an important role in both innate immune and adaptive immune. It was a key mediator in Th2-associated inflammatory disorders [29]. IL-33/ST2 axis is involved in the pathogenesis of liver injuries, such as viral hepatitis, acute hepatitis, and fatty liver disease [30]. Our data showed that IL-33 significantly decreased in infected mice with HPCs expansion and increased when the HPCs proliferation was inhibited. Knocking out of IL-33 could decrease the liver injury significantly in mice with few HPCs. Thus, we concluded that HPCs weakened the liver injury by repressing IL-33 secretion.

Our team has previously clarified that IL-33 contributed to schistosomiasis via inducing M2 macrophages polarization [31]. Pan and his colleagues reported that miR-203-3P from the liver promoted the *S. japonicum*-induced hepatic pathology by targeting IL-33 in HSCs [32]. In our study, we found that HPCs inhibit inflammatory granulomas and fibrosis and promote liver regeneration by repressing IL-33 secretion. Considering that HSCs and macrophages are two main cell components contributing to hepatic schistosomiasis [3], we analyzed the IL-33 in both of these two cell types. Interestingly, it displayed that the proliferation of HPCs negatively affected the expression of IL-33 in HSCs but not in macrophages. This reconfirmed that there is an interaction between HPCs and HSCs [33], which should be further investigated. All these results suggested that HPCs modulated the liver histopathology partly through modulating IL-33 secretion.

## Conclusions

Taken together, we found that HPCs mediated liver regeneration and inhibited IL-33 expression in hepatic schistosomiasis. These functions of HPCs played a protective role in liver injury induced by *S. japonicum* infection. Our study supplies a basic framework for further study of the therapeutic benefits of HPCs in the treatment of schistosomiasis.

## Supplementary Information

**Additional file 1**. Generation of IL-33^-/-^ mice by CRISPR/Cas9 technology. (A) The strategy of IL-33^-/-^ mice was generated by targeting the exon 3 region. (B) The genotype of IL-33^-/-^ mice was detected by PCR. The first lane named M is DNA marker. The amplification band of wild type is longer than 500bp and IL-33 knock out genotype is 500bp. (C) Genetic sequencing of purified PCR products of wild-type and IL-33^-/-^ mice.

**Additional file 2**. Isolation of primary hepatocyte, HSCs, and macrophages by two-step digestion with collagenase in situ. (**A**) After centrifuging in 12% iodixanol solutiom, cells were collected between two layers of liquid. HSCs were purified by negative section with CD11b antibody. (**B**) After centrifuging in 18% iodixanol solutiom, cells were collected between two layers of liquid. Macrophages were purified by positive section with CD11b antibody. (**C**). The* albumin* mRNA level was detected by Real-time PCR in primary hepatocytes, HSCs and macrophages. (**D**). The* α-SMA* mRNA level was detected by Real-time PCR in primary hepatocyte, HSCs and macrophages.

**Additional file 3**. The proliferation of HPCs didn’t affect the proliferated ability of mature hepatocytes in *S. japonicum* infection mice. Representative images of Ki67 staining.

## Data Availability

All the data used to support the findings of this study are included in this manuscript, and the original files can be available from the corresponding author upon reasonable request.

## Abbreviations

*S.**japonicum*: *Schistosoma japonicum*
HPCs: Hepatic progenitor cells
TWEAK: Tumor necrosis factor-like-weak inducer of apoptosis
HSCs: Hepatic stellate cells
ECM: Extracellular matrix
BSL-2: Biosafety level-2
*O.**hupehensis*: *Oncomelania hupensis*
ELISA: Enzyme-linked immunosorbent assay
H&E: Hematoxylin and eosin
IHC: Immunohistochemistry
CK19: Cytokeratin 19
Epcam: Epidermal cell adhesion molecule
HGF: Hepatocyte growth factor
TNF: Tumor necrosis factor
TGF: Transforming growth factor
