# Supplementary material for: Hepatic progenitor cells promote the repair of schistosomiasis liver injury by inhibiting IL-33 secretion in mice
Source: Stem Cell Res Ther. 2021 Oct 21;12:546. doi: 10.1186/s13287-021-02589-y (PMC8529826; doi:10.1186/s13287-021-02589-y)
Supplement: Supplementary file 1 — Additional file 1. Generation of IL-33-/- mice by CRISPR/Cas9 technology. (A) The strategy of IL-33-/- mice was generated by targeting the exon 3 region. (B) The genotype of IL-33-/- mice was detected by PCR. The first lane named M is DNA marker. The amplification band of wild type is longer than 500bp and IL-33 knock out genotype is 500bp. (C) Genetic sequencing of purified PCR products of wild-type and IL-33-/- mice. [file 13287_2021_2589_MOESM1_ESM.docx]

**Additional file 1**


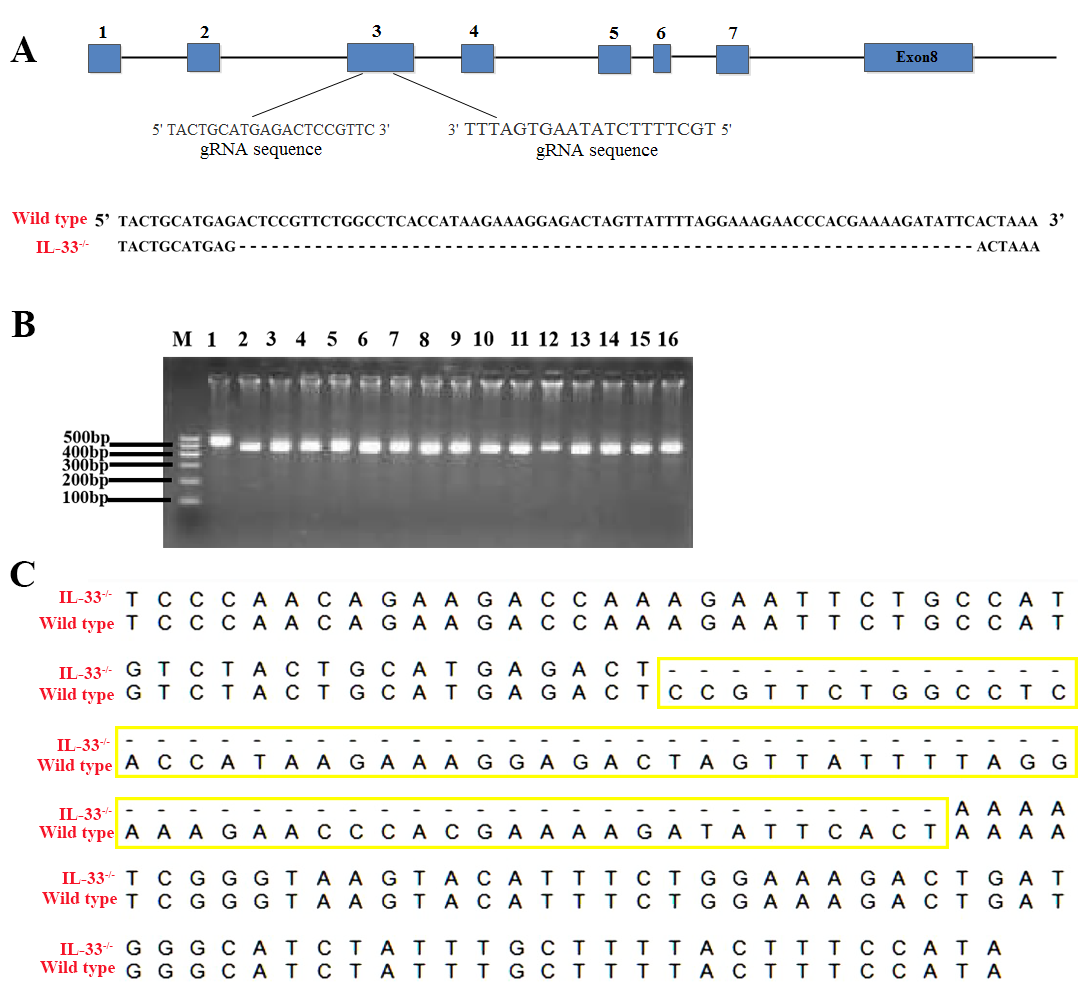


**Additional file 1:** **Generation of IL-33^-/-^ mice by CRISPR/Cas9 tecnology**. (A) The strategy of IL-33^-/-^ mice was generated by targeting the exon 3 region. (B) The genotype of IL-33^-/-^ mice was detected by PCR. The first lane named M is DNA marker. The amplification band of wild type is longer than 500bp and IL-33 knock out genotype is 500bp. (C) Genetic sequencing of purified PCR products of wild type and IL-33^-/-^ mice.
