# Supplementary material for: Hepatic progenitor cells promote the repair of schistosomiasis liver injury by inhibiting IL-33 secretion in mice
Source: Stem Cell Res Ther. 2021 Oct 21;12:546. doi: 10.1186/s13287-021-02589-y (PMC8529826; doi:10.1186/s13287-021-02589-y)
Supplement: Supplementary file 2 — Additional file 2. Isolation of primary hepatocyte, HSCs, and macrophages by two-step digestion with collagenase in situ. (A) After centrifuging in 12% iodixanol solutiom, cells were collected between two layers of liquid. HSCs were purified by negative section with CD11b antibody. (B) After centrifuging in 18% iodixanol solutiom, cells were collected between two layers of liquid. Macrophages were purified by positive section with CD11b antibody. (C). The albumin mRNA level was detected by Real-time PCR in primary hepatocytes, HSCs and macrophages. (D). The α-SMA mRNA level was detected by Real-time PCR in primary hepatocyte, HSCs and macrophages. [file 13287_2021_2589_MOESM2_ESM.docx]

**Additional file 2**


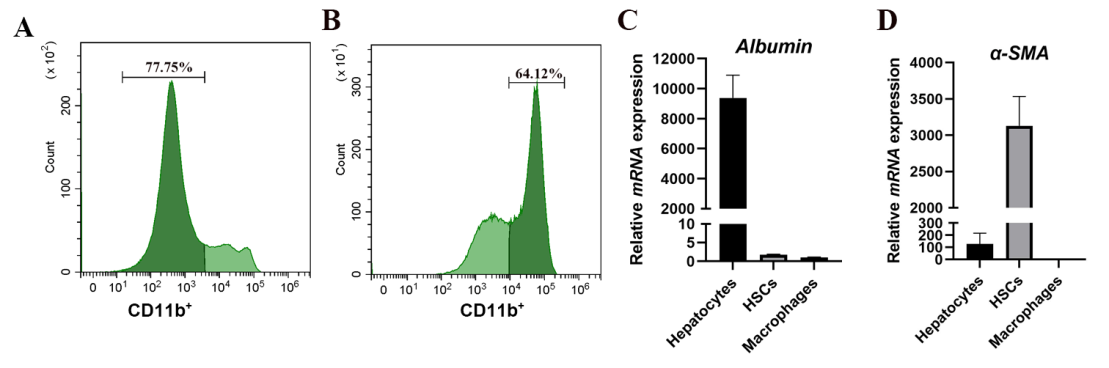


**Additional file 2: Isolation of primary hepatocyte, HSCs and macrophages by two-step digestion with collagenase in situ.** (A) After centrifuging in 12% iodixanol solutiom, cells were collected between two layers of liquid. HSCs were purified by negative section with CD11b antibody. (B) After centrifuging in 18% iodixanol solutiom, cells were collected between two layers of liquid. Macrophages were purified by positive section with CD11b antibody. (C). The *albumin* mRNA level was detected by Real-time PCR in primary hepatocyte, HSCs and macrophages. (D). The *α-SMA* mRNA level was detected by Real-time PCR in primary hepatocyte, HSCs and macrophages.
