# Supplementary material for: Hepatic progenitor cells promote the repair of schistosomiasis liver injury by inhibiting IL-33 secretion in mice
Source: Stem Cell Res Ther. 2021 Oct 21;12:546. doi: 10.1186/s13287-021-02589-y (PMC8529826; doi:10.1186/s13287-021-02589-y)
Supplement: Supplementary file 3 — Additional file 3. The proliferation of HPCs didn’t affect the proliferated ability of mature hepatocytes in S. japonicum infection mice. Representative images of Ki67 staining. [file 13287_2021_2589_MOESM3_ESM.docx]

**Additional file 3**


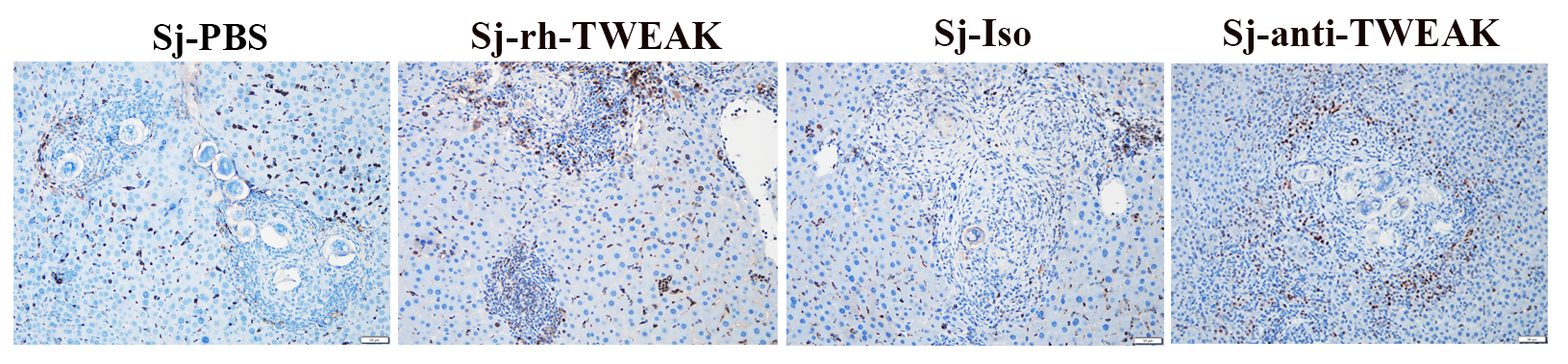


**Additional file 3: The proliferation of HPCs didn’t affect the proliferated ability of mature hepatocytes in *S. japonicum* infection mice**. Representative images of Ki67 staining.
